# Analytical Method for the Optimization of the Open-Hole and Filled-Hole Laminates at the Preliminary Design Stage

**DOI:** 10.3390/ma16062213

**Published:** 2023-03-09

**Authors:** Zubair Sajid, Saravanan Karuppanan, Kee Kok Eng, Syed Zulfiqar Hussain Shah

**Affiliations:** 1 Department of Mechanical Engineering, College of Electrical and Mechanical Engineering, National University of Sciences and Technology, Islamabad 44000, Pakistan; 2 Mechanical Engineering Department, Universiti Teknologi PETRONAS, Seri Iskandar 32610, Perak, Malaysia

**Keywords:** open-hole and filled-hole laminates, classical laminate theory, Lekhnitskii solutions, load-bearing capacity

## Abstract

In recent years, there has been an increasing interest in open-hole and filled-hole laminate failure analysis. The open and filled-hole laminate failure analysis is used in several important areas, especially in designing mechanically fastened composite joints. Various analytical, empirical, and numerical methods are available for the design of mechanically fastened composite joints. The large number of material and geometrical design variables at the preliminary design stage makes the empirical and numerical methods effortful, expensive, and time-consuming. Therefore, analytical methods are recommended over numerical and empirical methods at the preliminary design stage merely because of their simplification in calculations, making them computationally efficient. Taking this into consideration, current research presents an improvement to the analysis capabilities of the previously introduced analytical method, i.e., the coupled approach of Classical laminate theory (CLT) and Lekhnitskii solutions. These improvements include the development of failure envelops for the open-hole and filled-hole laminates, estimation of optimized filling material for attaining maximum load-bearing capacity of filled-hole laminates, and optimization of stacking sequence for maximum load-bearing capacity of open-hole and filled-hole laminates. From the failure envelop results, it was found that failure envelopes of filled-hole laminates are bigger than open-hole laminates. Furthermore, it was found that the stiffness of the filling material should be equal to the stiffness of the laminate to achieve maximum bearing strength of the filled-hole laminate. It was also demonstrated that the coupled approach of CLT and Lekhnitskii solutions may provide carpet plots that can be utilized to optimize the stacking sequence for open-hole and filled-hole laminates.

## Highlights

- A simplified analytical procedure was presented for the preliminary design of composite structures with open and filled holes.
- Filling material stiffness plays a crucial role in enhancing the bearing performance of filled-hole laminates.
- The CLT-Lekhnitskii coupled theory can generate carpet plots, which is useful for the stacking sequence optimization of open-hole and filled-hole composite structures during the preliminary design stage.

## 1. Introduction

Fiber-reinforced polymer (FRP) composites are widely used in the fields of aerospace, automobiles, energy, and civil engineering owing to their lightweight, high strength, superior fatigue, and durability [1,2,3,4,5]. For the joining of structural components and to give access to the interior of the structure, notched composite panels are widely employed [6]. These composite panels significantly reduce the weight and part count, consequently enhancing the aircraft’s efficiency [7,8,9,10,11,12,13,14]. Moreover, composite laminates are frequently employed in load-bearing structures [15]. It is important to investigate the allowable design values of composite structures, by considering various damages and defects [16]. To give engineers important design information, such as the strengths of composite laminates with cut-outs and defects, open-hole tension (OHT) and open-hole compression (OHC) tests are frequently performed. Because of these reasons, the open-hole tensile and open-hole compressive behavior of composite laminates has been the subject of extensive research in recent decades [17,18,19]. Nonetheless, predicting the behavior of notched composite laminates under various load conditions remains challenging [20,21,22].

In addition to OHT and OHC studies, it is important to predict the failure strength of filled-hole laminates under tensile loading because fasteners are extensively used for joining composite structures [23,24,25,26,27,28]. Yan et al. compared open-hole and filled-hole strength under varying ply orientations, washer sizes, and clamping loads [29]. According to their findings, the strength of the filled-hole specimen could be higher or lower than the strength of the open-hole specimens, depending on the size of the washer and failure mode. Open-hole test results cannot be applied to the filled-hole specimens, making filled-hole strength prediction just as significant as open-hole results. Most of the literature on open holes and filled holes used detailed modeling, which is not recommended during the preliminary design stages.

Empirical methods are difficult and expensive at the preliminary design stage due to a large number of geometrical and material design factors. Therefore, numerical and analytical tools are considered feasible methods for the preliminary designing of open-hole and filled-hole composite structures. However, FEA has the disadvantage of being computationally inefficient. In addition, progressive damage modeling has been used for the analysis of composite structures. Progressive damage models encompass complex failure criteria and material degradation models. On the other hand, analytical approaches use simplified methods, unlike numerical methods. Therefore, analytical methods are recommended over numerical methods at the preliminary design stage, merely because of their simplifications and time efficiency. By keeping this in consideration, an analytical approach was chosen in this paper to study the response of open and filled-hole laminates working under various scenarios.

Analytical solutions to predict the stress distribution around the circular hole is complex due to the presence of stress singularity. Therefore, several analytical models have been proposed to predict stress and strain distribution along the periphery of the circular and elliptical holes. Most of the contributions to the development of these models are from Muschelisvili [30], Savin [31], and Lekhnitskii [32]. However, researchers have shown an increased interest in using the Lekhnitskii solution. This is because the methodology proposed by Lekhnitskii gives more freedom to the researchers from the implementation point of view.

The analytical model developed by Lekhnitskii for isotropic plates was further improved and extended to orthotropic plates by Theocaris and Philippidis [33]. In addition, Nuismer and Whitney [34] proposed a failure criterion based on point and average stress to predict the notched strength of fiber-reinforced composites (FRC). Stresses used in this failure model are obtained from the Lekhnitskii solution. The main limitation of this approach lies in the assumption that FRC is assumed to behave linearly. As for elliptical holes, Tan [35,36] predicted the residual strength of FRC with an elliptic hole. The author used the Lekhnitskii solution to obtain stress distribution around the hole, which was then used to determine the notched strength. The same approach was used to predict the strength in glass/epoxy and graphite/epoxy FRC with inclined holes [37,38]. Regarding the coupling of the Lekhnitskii solution with failure criteria, Whitworth and Mahase [39] proposed a framework for predicting the damage location near the hole. Similarly, Chang et al. [40] used Yamada-Sun failure criteria [41] to predict the strength of a plate with multiple holes. Again, stress distribution along the periphery of the holes was obtained using the Lekhnitskii solution. As mentioned above, many researchers used Lekhnitskii solutions and coupled Lekhnitskii solutions with their theories to investigate the effects of different parameters. Zheng and Xu [42] used the same approach to understand the effect of hole geometry on stress distribution around the hole. Similarly, Hufenbach and Zhou [43] investigated the effect of elastic inclusion on stress distribution, and Gruber et al. [44] studied the stress distribution around the hole while assuming FRC as a finite plate, unlike the Lekhnitskii solution’s assumption.

Most of the analytical solutions used to study the effect of inclusions are based on coupled theories. These theories use classical laminate theory (CLT) and complex potential functions. Hence, it can predict the coupling effect of multilayered unsymmetrical composites. A few authors coupled CLT and Lekhnitskii solutions to predict failure in FRC with open inclusions. Bradshaw and Pang [45] used CLT and Tsai-Wu failure criteria to predict failure in composite with a circular hole. Similarly, Cheng and Chang [46] used CLT and Lekhnitskii solutions to predict interlaminar stresses in a circular hole under in-plane loadings.

All of these investigations have demonstrated that CLT can be coupled with the Lekhnitskii solution to predict the strength of open-hole laminates. Therefore, this research expands the analysis capabilities of this coupled theory, which would be very helpful for the designing of open-hole and filled-hole structures at the preliminary design stages. All previous research on CLT and the Lekhnitskii solution examined only the strength prediction capability of open-hole laminates. However, this research work presents the strength prediction of filled-hole laminates by using CLT-Lekhnitskii coupled theory. In addition, the current research revealed the capability of coupled theory to produce the failure envelopes of open-hole and filled-hole laminates, as well as its capability to generate carpet plots for the optimization of stacking sequence. These improvements in CLT-Lekhnitskii coupled theory have not been reported in the literature.

## 2. Material Used and Laminate Sequence

Recently, most industries have started implementing end-of-life management in their products. Two factors need to be considered to achieve end-of-life management, namely recyclability and environmental friendliness of the product. Therefore, the material must have these two characteristics [47]. Considering the end-of-life management of the products, most industries have replaced conventional composite materials with green materials. One of the best options is to use basalt to replace glass fibers. Basalt fiber is a type of high-performance inorganic fiber produced from natural basalt. In comparison to glass, carbon, and other fibers, basalt fibers have very good characteristics at a reasonable price. In terms of performance, basalt fiber falls in between carbon fiber and glass fiber. Basalt fibers offer higher tensile strength than E-glass fibers, higher failure strain than carbon fibers, and good resistance to chemicals, impact load, and fire, see Table 1 [48,49]. Importantly, basalt is a green material and has excellent recyclability [50,51]. Therefore, the current research focuses on the basalt fiber open-hole and filled-hole laminates.

Huang and Xin [52] proposed a micromechanical approach to calculate the stiffness and strength of a laminate. For the micromechanical approach, only constituent-level properties are required. Equation (1) is used to calculate the stiffness properties of the laminates [53,54].

(1) $$\begin{array}{c} E_{1}=E_{11}^{f}V_{f}\;+\;E_{m}V_{m},\;E_{m}^{\prime }=\frac{E_{m}}{\left(1\;-\;V_{m}^{2}\right)}\\ \;E_{2}=\frac{E_{22}^{f}E_{m}^{\prime }}{E_{m}^{\prime }V_{f}\;+\;E_{22}^{f}V_{m}},\;G_{12}=\frac{1}{\left(\frac{V_{f}}{G_{12}^{f}}\;+\;\frac{V_{m}}{G_{m}}\right)} \end{array}$$

where $E_{1}$ is the elastic modulus of a ply in the longitudinal direction,$\;E_{2}$ is the elastic modulus of a ply in the transverse direction,$\;G_{12}$ is the in-plane shear modulus, $E_{11}^{f}$ is the longitudinal elastic modulus of fiber, $E_{22}^{f}$ is the transverse elastic modulus of fiber,$\;G_{12}^{f}$ is the fiber shear modulus, $E_{m}$ is the elastic modulus of the matrix, $\;E_{m}^{\prime }$ is the modified elastic modulus of the matrix, $G_{m}$ is the matrix shear modulus, $V_{f}$ is the fiber volume fraction, $V_{m}$ is the matrix volume fraction.

Equation (2) shows the relations used for the calculation of the longitudinal strength of laminates [52].

(2) $$\begin{array}{c} \sigma_{11}^{u,t}=\frac{\left(V_{f}E_{11}^{f}\;+\;V_{m}E^{m}\right)\sigma_{u,t}^{f}}{E_{11}^{f}},\;\sigma_{11}^{u,c}=\frac{\left(V_{f}E_{11}^{f}\;+\;V_{m}E^{m}\right)\sigma_{u,c}^{f}}{E_{11}^{f}},\;\\ \sigma_{12}^{u}=\frac{\left[\left(V_{f}\;+\;0.45V_{m}\right)G_{12}^{f}\;+\;0.55V_{m}G^{m}\right]\sigma_{u,s}^{m}}{0.45G_{12}^{f}\;+\;0.55G^{m}} \end{array}$$

where $\sigma_{11}^{u,t}$ is the tensile strength of a ply in the longitudinal direction,$\;\sigma_{u,t}^{f}$ is the fiber tensile strength, $\sigma_{11}^{u,c}$ is the compressive strength of a ply in the longitudinal direction, $\sigma_{u,c}^{f}$ is the fiber compressive strength,$\;\sigma_{12}^{u}$ is the in-plane shear strength of a ply, $\sigma_{u,s}^{m}$ is the matrix shear strength.

For the calculation of transverse strength, Equation (3) was used [52].

(3) $$\begin{array}{c} \sigma_{22}^{u,t}=\frac{\left[\left(V_{f}\;+\;0.4V_{m}\right)E_{22}^{f}\;+\;0.6V_{m}E^{m}\right]\sigma_{u,t}^{m}}{\left(0.4E_{22}^{f}\;+\;0.6E^{m}\right)K_{22}^{t}},\\ \sigma_{22}^{u,c}=\frac{\left[\left(V_{f}\;+\;0.4V_{m}\right)E_{22}^{f}\;+\;0.6V_{m}E^{m}\right]\sigma_{u,c}^{m}}{\left(0.4E_{22}^{f}\;+\;0.6E^{m}\right)K_{22}^{c}},\\ \sigma_{23}^{u}=\frac{\left[\left(V_{f}\;+\;0.4V_{m}\right)E_{22}^{f}\;+\;0.6V_{m}E^{m}\right]\sigma_{u,s}^{m}}{\left(0.4E_{22}^{f}\;+\;0.6E^{m}\right)K_{23}} \end{array}$$

where $K_{22}^{t}$, $K_{22}^{c}$, and $K_{23}$ are the stress concentration factors (SCF) of the matrix in a unidirectional composite subjected to transverse tension, transverse compression, and shear load, respectively. $K_{22}^{t}$, $K_{22}^{c}$ and $K_{23}$ can be calculated by using Equations (4)–(6).

(4) $$K_{22}^{t}=\begin{array}{l} \left[1\;+\;\frac{\sqrt{V_{f}}}{2}A\;+\;\frac{\sqrt{V_{f}}}{2}\left(3\;-\;V_{f}\;-\;\sqrt{V_{f}}\right)B\right]\\ \times \left[\frac{\left(V_{f}\;+\;V_{m}\beta \right)E_{22}^{f}\;+\;V_{m}\left(1\;-\;\beta \right)E^{m}}{\beta E_{22}^{f}\;+\;\left(1\;-\;\beta \right)E^{m}}\right] \end{array}$$

(5) $$\begin{array}{c} K_{22}^{c}=\{1\;-\;\frac{\sqrt{V_{f}}}{2}A\frac{\sigma_{u,c}^{m}\;-\;\sigma_{u,t}^{m}}{\sigma_{u,c}^{m}\;+\;\sigma_{u,t}^{m}}\\ \;+\;\frac{B}{2\left(1\;-\;\sqrt{V_{f}}\right)}\{-V_{f}^{2}\left[1\;-\;2{\left(\frac{\sigma_{u,c}^{m}\;-\;\sigma_{u,t}^{m}}{\sigma_{u,c}^{m}\;+\;\sigma_{u,t}^{m}}\right)}^{2}\right]\\ \;+\;\frac{4\sigma_{u,t}^{m}V_{f}}{\sigma_{u,c}^{m}\;+\;\sigma_{u,t}^{m}}\left(1\;+\;2\frac{\sigma_{u,c}^{m}\;-\;\sigma_{u,t}^{m}}{\sigma_{u,c}^{m}\;+\;\sigma_{u,t}^{m}}\right)\\ -\sqrt{V_{f}}\left[2\frac{\sigma_{u,c}^{m}\;-\;\sigma_{u,t}^{m}}{\sigma_{u,c}^{m}\;+\;\sigma_{u,t}^{m}}\;+\;1\;-\;2{\left(\frac{\sigma_{u,c}^{m}\;-\;\sigma_{u,t}^{m}}{\sigma_{u,c}^{m}\;+\;\sigma_{u,t}^{m}}\right)}^{2}\right]\}\}\\ \times \left[\frac{\left(V_{f}\;+\;V_{m}\beta \right)E_{22}^{f}\;+\;V_{m}\left(1\;-\;\beta \right)E^{m}}{\beta E_{22}^{f}\;+\;\left(1\;-\;\beta \right)E^{m}}\right] \end{array}$$

(6) $$K_{23}=\sigma_{u,s}^{m}\left[\frac{K_{22}^{t}}{\sigma_{u,t}^{m}}\;+\;\frac{K_{22}^{c}}{\sigma_{u,c}^{m}}\right]$$

where $\sigma_{u,c}^{m}$ is the matrix compressive strength and $\sigma_{u,t}^{m}$ is the matrix tensile strength. The values of A and B can be calculated by using Equations (7) and (8).

(7) $$A=\frac{\left[1\;-\;v^{m}\;-\;2{\left(v^{m}\right)}^{2}\right]E_{22}^{f}\;-\;\left[1\;-\;v_{23}^{f}\;-\;2{\left(v_{23}^{f}\right)}^{2}\right]E^{m}}{\left(1\;+\;v^{m}\right)E_{22}^{f}\;+\;\left[1\;-\;v_{23}^{f}\;-\;2{\left(v_{23}^{f}\right)}^{2}\right]E^{m}}$$

(8) $$B=\frac{\left(1\;+\;v_{23}^{f}\right)E^{m}\;-\;\left(1\;+\;v^{m}\right)E_{22}^{f}}{\left[v^{m}\;+\;4{\left(v^{m}\right)}^{2}\;-\;3\right]E_{22}^{f}\;-\;\left(1\;+\;v_{23}^{f}\right)E^{m}}$$

Based on this micromechanical approach (Equation (1) to Equation (3)), the strength and stiffness properties of the basalt laminate were estimated and tabulated in Table 2. X_T_, X_C_, Y_T_, and Y_C_ are longitudinal strength in tension, longitudinal strength in compression, transverse strength in tension, and transverse strength in compression, respectively. Similarly, E_11_, E_22_, G_12_, G_23_, *v*_12,_ and *v*_23_ are longitudinal stiffness, transverse stiffness, in-plane shear stiffness, out-of-plane shear stiffness, in-plane Poisson’s ratio, and out-of-plane Poisson’s ratio, respectively. Constituent properties of basalt fiber and matrix [55] are listed in Table 2. The ply properties are estimated using a 60% fiber volume fraction. The basic idea is that higher fiber volume fractions are required for primary structural applications, and thanks to the advancements in composite manufacturing techniques such as vacuum resin infusion, higher volume fractions of 50% to 60% can be easily achieved for unidirectional laminates. Therefore, a fiber volume fraction of 60% is used in this article so that the results presented can be related to primary structural applications. Data obtained in Table 2 was then used in CLT-Lekhnitskii coupled theory for the prediction of bearing strength.

Different laminate sequences were considered in this study. In the aerospace industry, only 0°, 45°, and 90° plies are used. Furthermore, composite products used in the aerospace industry must follow the 10% rule [56]. According to this rule, the laminate must consist of a minimum of 10% of each ply. Therefore, three laminate sequences were considered to study the effect of laminate sequence on the performance of open and filled-hole laminates. These sequences are soft: ${\left[0^{{}^{\circ}}/45^{{}^{\circ}}/90^{{}^{\circ}}{}_{4}/-45^{{}^{\circ}}/0^{{}^{\circ}}\right]}_{s},$ hard: ${\left[90^{{}^{\circ}}/45^{{}^{\circ}}/0^{{}^{\circ}}{}_{4}/-45^{{}^{\circ}}/90^{{}^{\circ}}\right]}_{s}$ and QI: ${\left[0^{0}/\pm 45^{0}/90^{0}\right]}_{2s}$. Soft laminates mostly consist of off-axis plies [57], whereas hard laminates consist mostly of on-axis plies. QI laminates share an equal amount of each ply [58].

## 3. Analytical Modeling

This section aims to describe the methodology used in this study. CLT [59] is considered a fundamental analytical approach to studying the response of laminates. However, it could only be used for un-notched laminates. This was considered the main limitation of using CLT alone for notched laminates. As discussed earlier, some researchers have shown the compatibility of CLT with Lekhnitskii [32] solutions. Therefore, the methodological approach taken in this study is a mixed methodology based on the combination of CLT and Lekhnitskii solutions. The combination of these approaches is beneficial in studying the response of open and filled-hole laminates. The effective material properties of a laminate were calculated first. “A” matrix was required from CLT to calculate effective material properties. Effective material properties were estimated by using Equation (9).

(9) $$E_{1}\;=\;\frac{A_{11}^{-1}}{h},\;E_{2\;}=\frac{A_{22}^{-1}}{h},S_{12}=\;\frac{A_{33}^{-1}}{h},v_{1}=\;\frac{A_{12}^{-1}}{A_{11}^{-1}}$$

The next step was to calculate constants k, n,$\;a_{1},$ $a_{2},\;a_{3},\;a_{4},\;∆,\;\mathrm{and}\;E_{\theta }$, which were used to calculate σ_r_, σ_t_, and σ_rt_. Equation (10) was utilized to estimate the value of $E_{\theta }$.

(10) $$\frac{1}{E_{\theta }}=\frac{sin^{4}\theta }{E_{1}}\;+\;\left(\frac{1}{G}\;-\;\frac{2v_{1}}{E_{1}}\right)sin^{2}\theta cos^{2}\theta \;+\;\frac{cos^{4}\theta }{E_{2}}$$

**Figure 1:**
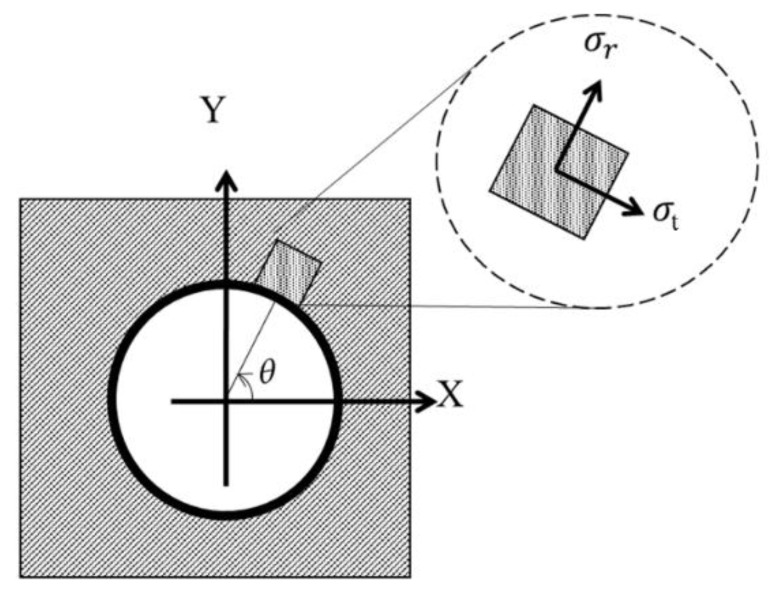
Definition of laminate stresses.

Laminate stresses are described in Figure 1. Equations (11) to (13) were used to calculate these stresses. $E_{\theta },\sigma$_r_, σ_t_, and σ_rt_ were calculated for each angle θ.

(11) $$\sigma_{r}=\frac{p}{2∆}\left[∆\;+\;a_{3}\;+\;a_{2}\;+\;\left(∆\;+\;a_{3}\;-\;a_{2}\right)cos2\theta \right]$$

(12) $$\sigma_{rt}=\frac{-p}{2∆}\left(∆\;+\;a_{3}\;-\;a_{2}\right)sin2\theta$$

(13) $$\sigma_{t}=\frac{p}{∆}\frac{E_{\theta }}{E_{1}}\left\{\left(∆\;-\;a_{1}\right)sin^{4}\theta \;+\;\left[a\right]sin^{4}\theta cos^{2}\theta \;+\;\left[b\right]sin^{2}\theta cos^{4}\theta \;-\;k^{2}a_{4}cos^{6}\theta \right\}$$

where, a = $∆\left(n^{2}\;-\;2k\right)\;+\;\left(k\;+\;n\right)a_{1}\;+\;\left(1\;+\;2k\right)a_{2}\;-\;\left(2\;+\;k\right)\left(1\;+\;n\right)a_{3}$.

b = $k^{2}∆\;-\;\left(1\;+\;2k\right)\left(k\;+\;n\right)a_{2}\;+\;k\left(2\;+\;k\right)a_{3}\;+\;k\left(1\;+\;n\right)a_{4}$.

After calculating the laminate stresses, the transformation matrix was used to get the global loads, as given in Equation (14).

(14) $$\left(\begin{array}{c} N_{1}\\ N_{2}\\ N_{3} \end{array}\right)=\left[\begin{array}{c} cos^{2}\theta \\ sin^{2}\theta \\ cos\theta sin\theta \end{array}\begin{array}{c} sin^{2}\theta \;\\ cos^{2}\theta \\ -cos\theta sin\theta \; \end{array}\begin{array}{c} -2cos\theta sin\theta \\ 2cos\theta sin\theta \\ cos^{2}\theta \;-\;sin^{2}\theta \end{array}\right]\left(\begin{array}{c} h\sigma_{r}\\ h\sigma_{t}\\ h\sigma_{rt} \end{array}\right)$$

Once the global loads were evaluated, CLT was implemented to assess the failure index along the periphery of the hole. Hashin failure criteria [52] were employed to predict the failure index of the laminates. Equations (15) to (18) listed different failure modes, namely fiber failure in tension (FFT), fiber failure in compression (FFC), matrix failure in tension (MFT), and matrix failure in compression (MFC), respectively. Failure index using Equation (15) to Equation (18), is calculated along the whole periphery of the hole, i.e., 0° to 360°, and among all the 360 values, only the maximum value of failure index is selected to calculate the bearing strength.

(15) $$FFT=\frac{\sigma_{11}^{2}}{X_{t}^{2}}\;+\;\frac{\sigma_{12}^{2}}{S^{2}}$$

(16) $$FFC=\frac{-\sigma_{11}}{X_{t}}$$

(17) $$MFT=\frac{\sigma_{22}^{2}}{Y_{t}^{2}}\;+\;\frac{\sigma_{12}^{2}}{S^{2}}$$

(18) $$MFC=\frac{\sigma_{22}^{2}}{4S^{2}}\;+\;\left[{\left(\frac{Y_{C}}{2S}\right)}^{2}\;-\;1\right]\frac{\sigma_{12}}{Y_{C}}\;+\;\frac{\sigma_{12}^{2}}{S^{2}}$$

**Figure 2:**
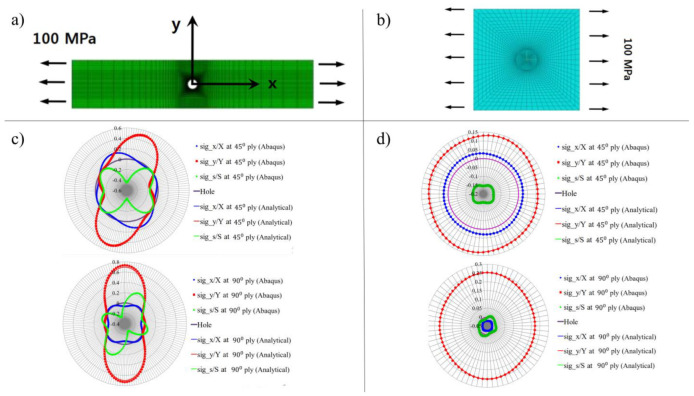
Validation of proposed analytical model with finite element model: (**a**) FEM model of open-hole laminate (**b**) FEM model of filled-hole laminate (**c**) Stress to strength ratio comparison of 45° and 90° plies in open-hole laminate (**d**) Stress to strength ratio comparison of 45° and 90° plies in filled-hole laminate.

The proposed analytical model was validated with the finite element model in Abaqus. Stacking sequence ${\left[0/+45/-45/90\right]}_{s}$ was used for validation purposes. Figure 2a,b shows the FEM models of open-hole and filled-hole laminates, which were generated in Abaqus. Figure 2c,d compares the longitudinal stress to longitudinal strength ratio, i.e., sig_x/X, transverse stress to transverse strength ratio, i.e., sig_y/Y, and shear stress to shear strength ratio, i.e., sig_s/S, of Abaqus and analytical model. The results of the Abaqus model were determined at the periphery of the hole and compared with the analytical model. It was found that the results generated by the analytical model matched well with the Abaqus model.

**Figure 3:**
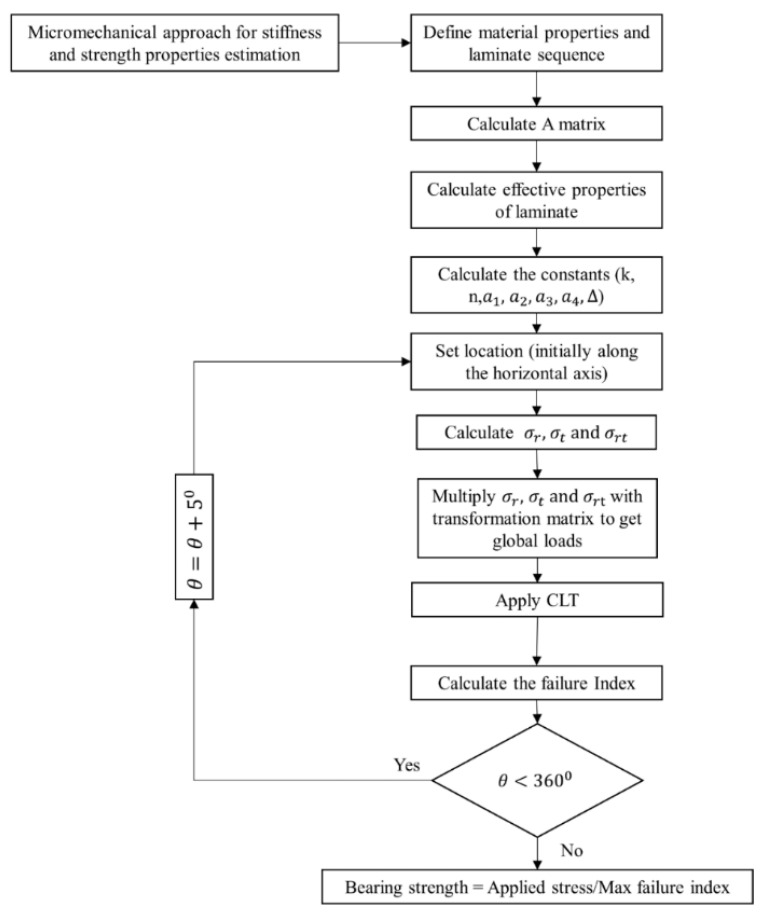
Flow chart of the analytical procedure.

**Figure 4:**
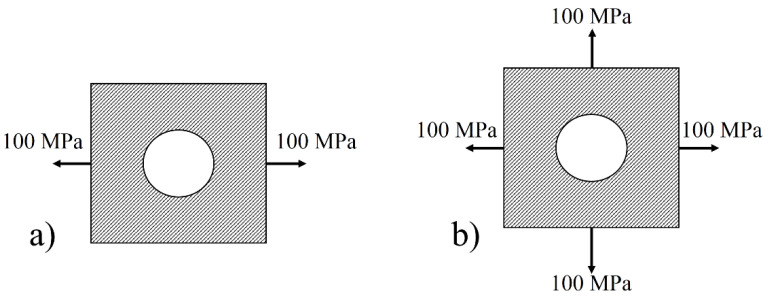
Boundary conditions for each loading case (**a**) Uniaxial loadings (**b**) Multiaxial loadings.

Figure 3 shows the flow chart of the procedure mentioned above. The current research aims to achieve the maximum load-bearing capacity of open and filled-hole laminates made up of basalt fibers, subjected to different loading conditions. Different loading cases are indicated in Figure 4. Loading is divided into two main groups, namely uniaxial and multiaxial loading. 100 MPa load was applied along the x-axis in the uniaxial loading case. Meanwhile, an equal amount of loading was applied along the x-axis and y-axis in the multiaxial loading case. 

## 4. Results and Discussion

The primary purpose of the current research is to extend further the capability of the CLT-Lekhnitskii coupled theory by adding three modules, i.e., strength prediction of open-hole and filled-hole laminates, failure envelopes of open-hole and filled-hole laminates and carpet plots for prediction of stacking sequence. The results of these three modules are discussed below.

### 4.1. Strain-Based Failure Envelops

Failure of laminates depends on many factors, and a few of them are focused on in this study. These factors were the stiffness of the filling material, laminate sequence, and loading conditions. To evaluate the factors mentioned above in a broader aspect, a failure envelope was developed for a better representation of failure boundaries under various loading conditions. Therefore, the current section describes the effect of laminate sequences and different filling materials on the failure envelop of the open-hole and filled-hole laminates.

**Figure 5:**
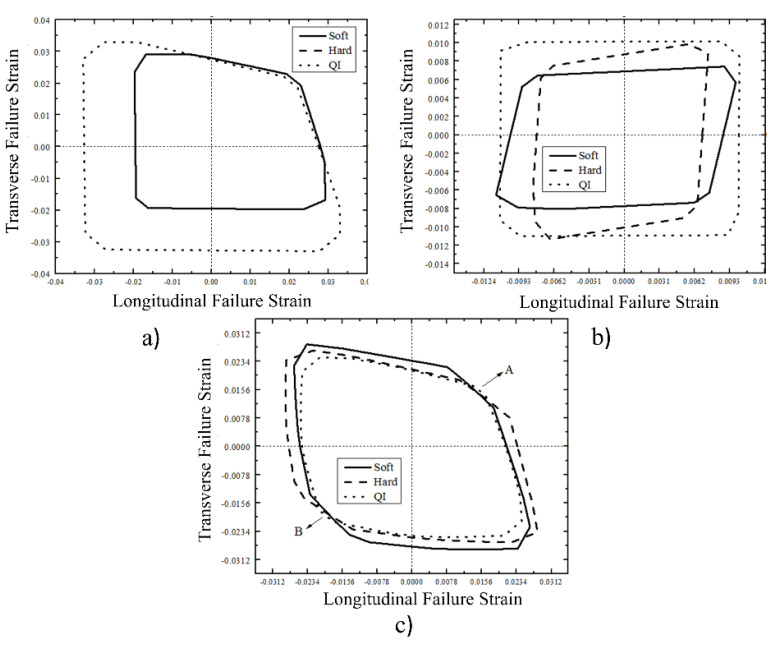
Comparison of failure envelops for different laminate sequences (**a**) Un-notched laminate (**b**) Open-hole laminate (**c**) Filled-hole laminate.

As shown in Figure 5a, a significant difference in size was found between QI un-notched laminates and hard and soft laminates. In addition, hard and soft laminates produce the same failure envelope. A possible explanation for this phenomenon is that all these calculations were done based on first-ply failure criteria and both of these laminates are experiencing the same first-ply failure, i.e., 0° or 90°. A microscopic study would give a more accurate reason. Despite the significant differences in the size of failure envelopes for different un-notched laminates, all three un-notched laminates exhibit the exact shape of failure envelopes. When shifting from un-notched to the open-hole case, failure envelopes of all the laminate sequences are reduced, as depicted in Figure 5b. The free edge stresses along the periphery of the hole cause a reduction in failure envelopes. Furthermore, the failure envelope of QI open-hole laminate was rectangular but still bigger than hard and soft open-hole laminates. This concludes that QI open-hole laminate has higher failure strains in all the loading directions. Unlike un-notched laminates, the shape of hard and soft open-hole laminates differs. It was observed that QI laminates performed well for un-notched and open-hole laminates.

As far as filled-hole laminates are concerned, a further change in the shape of failure envelopes was recorded. By examining Figure 5c, higher values of failure strains were found compared to the open-hole case. This is because free edge stresses at the periphery of the hole are reduced due to the presence of the filling material. Further examination revealed that soft laminates performed well for transverse tension and compression, and hard laminates performed well for longitudinal tension and compression. This phenomenon is because many plies are in the loading direction for hard laminates, whereas in soft laminates, most of the plies are perpendicular to the loading. Interestingly, the failure envelopes of the QI filled-hole laminate are smaller than both soft and hard laminates. However, at points A and B, as indicated in Figure 5c, QI filled-hole laminates showed slightly higher failure strain than soft and hard laminates. These were the cases when an equal amount of tensile and compressive loadings was applied in both longitudinal and transverse directions. This concludes that, in filled-hole laminates, loading directions are essential along with the laminate sequence. This is because hard laminates showed higher failure strains in longitudinal directions, soft laminates showed higher failure strains in transverse directions, and QI laminates showed slightly higher failure strains when equal loadings were applied in both longitudinal and transverse directions.

### 4.2. Effect of Filling Material on Load Bearing Capacity

**Figure 6:**
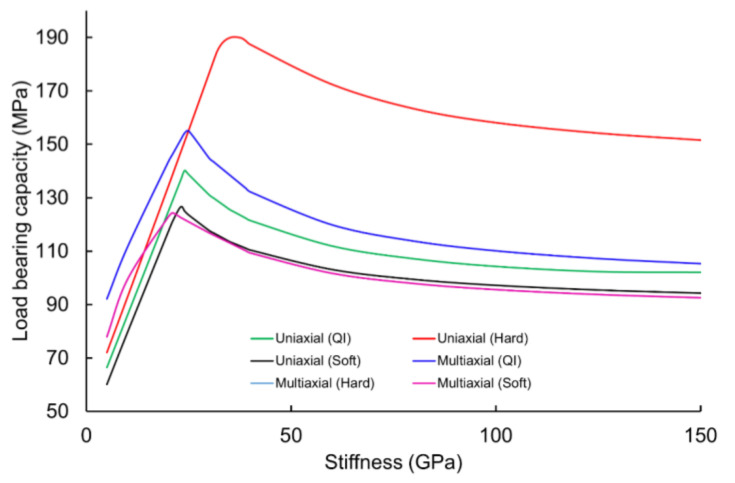
Variation of load-bearing capacity for different laminates subjected to uniaxial and multiaxial loading.

As discussed earlier, open-hole laminates have smaller failure envelopes than filled-hole laminates. Therefore, it can be said that open-hole laminates have the lowest load-bearing capacity. This low capacity was because of high-stress concentrations along the periphery of the hole. This high-stress concentration could be due to the free edge stresses. This stress concentration could be decreased by introducing filling material into the hole. The most prominent observation to emerge from the data comparison was the correlation between load-bearing capacity and stiffness of the filling material. The variation of load-bearing capacity with respect to the stiffness of the filling material is shown in Figure 6. It was found that the load-bearing capacity has a direct relationship with the stiffness of the filling material. With the filling material’s stiffness increase, load-bearing capacity is further increased. This was because of some load sharing by the filling material. However, this relation is true up to a particular value of stiffness of filling material. Afterward, load-bearing capacity decreases exponentially with an increase in the stiffness of the filling material. In other words, a further increase in the stiffness of the filling material adversely affects the situation. Consequently, a decrease in the load-bearing capacity of the laminate was found when the stiffness of the filling material increased beyond the optimal value, which is 24 GPa for uniaxial loadings of QI laminates. From Table 3, it was found that the longitudinal and transverse equivalent stiffnesses, i.e., E_1_ and E_2_, of the QI base laminate are also 24.1 Gpa. Therefore, it can be concluded that if the stiffness of the filling material is close to the equivalent stiffness of the base laminates, then the filling material can perform as a load-transferring agent. Whereas, if the stiffness of the filling material is higher than the equivalent stiffness of the base laminates, then the filling material imparts load on the wall of the hole which causes a reduction in the load-bearing capability.

This behavior remained the same for different laminate sequences under various loading conditions. However, the values of the maximum load-bearing capacity and their respective stiffnesses of the filling materials were different for different loading conditions and laminate sequences. As shown in Table 3, the stiffness values of the filling material varied from 21 Gpa to 36 Gpa, depending on the loading conditions and laminate sequence. The maximum value for the filling material was 36 Gpa for hard laminates under uniaxial loadings. The reason is that hard laminate contains large percentages of 0° plies in the loading direction. As shown in Table 3, to achieve maximum load-bearing capacity for QI laminates under both uniaxial and multiaxial loadings, the stiffness of the filling material should be the same as the stiffness of the base laminate. For the case of hard and soft laminates, optimum values of the filling material stiffness were found to be close to the stiffness of the base laminate. These optimum values of the filling material of hard and soft laminates depend on the *E*_1_ and *E*_2_ of the laminate. It was found that for hard laminates under uniaxial loadings, the stiffness of the filling material should be close to the *E*_1_ of the laminate. In addition, the stiffness of the filling material should be close to the *E*_2_ of the laminate if the hard laminates are subjected to multiaxial loadings. This is contrary to the case of the soft laminate, where the stiffness of the filling material should be close to the *E*_1_ of the base laminate when subjected to either uniaxial loadings or multiaxial loadings. It is important to note that the data points of multiaxial (hard) overlap the multiaxial (soft) data points, as shown in Figure 6. Based on the discussion above, it can be concluded that the decision of selecting the filling material stiffness can be made by considering the loading conditions and equivalent stiffness of the base laminates. For uniaxial loading conditions, the stiffness of the filling material should be equal to or close to the longitudinal equivalent stiffness of the base laminate, irrespective of the stacking sequence. Whereas, for multiaxial loading conditions, the stiffness of the filling material should be equal to or close to the transverse-equivalent stiffness of the base laminate, irrespective of the stacking sequence.

### 4.3. Effect of Laminate Sequence on Load Bearing Capacity

**Figure 7:**
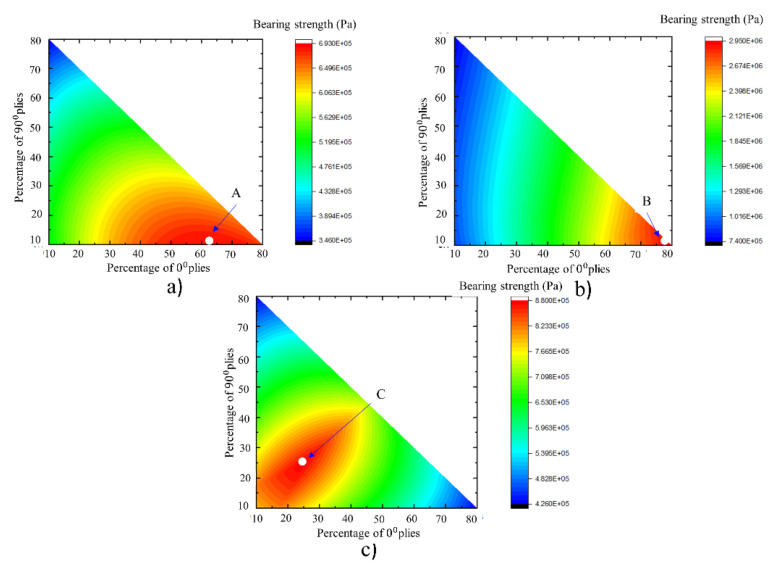
Carpet plots for load-bearing capacity (**a**) Open-hole laminate under uniaxial loadings (**b**) Filled-hole laminate under uniaxial loadings (**c**) Open-hole laminate under multiaxial loading.

The study of the failure envelopes revealed that the percentage usage of 0°, 45°, and 90° plies in a laminate is of paramount importance. By setting the amount of 0°, 45°, and 90° plies in a laminate, the maximum load-bearing capacity of open and filled-hole laminates can be achieved under different loading scenarios. Table 4 presents the recommended percentages of each ply in a laminate for maximum load-bearing capacity. These results were generated by keeping in mind the 10% rule discussed earlier. Contour plots in Figure 7 show the bearing strength values in Pascals. The red color demonstrates the larger values of the bearing strengths while the blue color shows the smaller values of the bearing strengths. It is clear from Figure 7a,b that a higher percentage of 0° plies is required for both open and filled-hole laminates under uniaxial loadings, as the red contours which are indicative of higher values of bearing strengths, are concentrated on the right of the x-axis. From Table 4, it was observed that the percentages of 0° and 45° plies were different for the open hole and filled hole laminates under uniaxial loadings. A higher number of 45° plies were used in open-hole laminates compared to the filled-hole laminates. Unlike filled-hole laminates, 45° plies also contribute to load sharing, along with 0° plies in open-hole laminates. As far as filled-hole laminates are concerned, all the filled-hole laminates have the same high percentage of 0° plies. It can therefore be assumed that the stiffness of the filling material does not affect the percentage of the plies in filled-hole laminates under uniaxial loadings (i.e., the same percentage of 0° plies in both aluminum-filled, and steel-filled laminates under uniaxial loadings).

On the other hand, the percentages of plies were different for multiaxial loading. As shown in Figure 7c, higher values of bearing strength can be achieved by keeping the fractions of 0° and 90° plies equal. However, the 45° plies fractions or percentage in a laminate should be higher than the combined fractions of 0 and 90° plies, as evident from Table 4 (point C). It can be hypothesized by evaluating Table 4 that percentages of the plies are insensitive to the open and filled-hole laminates under multiaxial loading.

## 5. Conclusions

The current research work aims to propose an analytical method for the preliminary design stage of open-hole and filled-hole composite structures that is capable of finalizing the choice of stacking sequence and filling material. For that purpose, a CLT-Lekhnikstii coupled approach was used to generate failure envelops and carpet plots, and this theory was also used to select the filler material. The proposed analytical tool was found to be very useful during the preliminary design stage, and the following key conclusions were reached using the CLT-Lekhnikstii coupled approach:

- The CLT-Lekhnikstii coupled approach can generate failure envelops for open-hole and filled-hole composite structures, which is a very beneficial finding for the preliminary design stage of these structures.
- To achieve maximum bearing strength, the stiffness of the filler material for joining two composite laminates should be equal to or close to the stiffness of the joining composite laminates. For uniaxial loading conditions, the stiffness of the filler material should be close to the longitudinal equivalent stiffness of the base laminate. Meanwhile, for multiaxial loading conditions, it should be close to the transverse-equivalent stiffness of the base laminate.
- Carpet plots produced by CLT-Lekhnikstii coupled method can be very effective for deciding the stacking sequence of open-hole and filled-hole composite structures at the preliminary design stage.

## Figures and Tables

**Table 1:** Material properties of different fiber types.

Fiber	$\mathrm{Density}\;(g/cm^{3})$	Failure Strain (%)	Tensile Strength (MPa)	Stiffness (GPa)
Basalt	2.63–2.8	3.1–6	3000–4840	93–110
E-glass	2.54–2.57	4.7	3100–3800	72.5–75.5
Carbon	1.78	1.5–2.0	3500–6000	230–600

**Table 2:** Material properties of basalt fabric.

Material Properties	Values
Stiffness properties (fiber)	E_11_ = E_22_ = 90 GPa, G_12_ = 35.70 GPa, *v*_12_ = *v*_23_ = 0.26
Strength properties (fiber)	X_T_ = X_C_ = 2950 MPa
Stiffness properties (Matrix)	E_11_ = 3.20 GPa, G_12_ = 1.18 GPa, *v*_12_ = 0.35
Strength properties (Matrix)	X_T_ = 73 MPa, X_C_ = 120 MPa, S = 52 MPa
Stiffness properties (Laminate)	E_11_ = 55.28 GPa, E_22_ = 8.95 GPa, G_12_ = 3.32 GPa, G_23_ = 2.70 GPa, *v*_12_ = 0.29, *v*_23_ = 0.40
Strength properties (Laminate)	X_T_ = X_C_ = 1811.95 MPa, Y_T_ = 49.29 MPa, Y_C_ = 127.97 MPa, S = 87.42 MPa

Fiber volume fraction = 0.6.

**Table 3:** Optimum values of the stiffnesses of the filling material for different laminate sequences subjected to uniaxial and multiaxial loading.

Laminate Seq.	Uniaxial Loadings (Gpa)	Multiaxial Loading (Gpa)	$\mathrm{Base}\;\mathrm{Laminate}\;\mathrm{Equivalent}\;\mathrm{Stiffness}\;E_{1}\;$ (Gpa)	$\mathrm{Base}\;\mathrm{Laminate}\;\mathrm{Equivalent}\;\mathrm{Stiffness}\;E_{2}\;$ (Gpa)
QI	24	24	24.1	24.1
Hard	36	21	34.2	22.9
Soft	23	21	22.9	34.2

**Table 4:** Recommended percentages of plies for maximum load capacity.

Laminate	Loading	0°	90°	45°
Open hole (A)	Uniaxial	63	10	27
Al. filled hole		80	10	10
St. filled hole (B)		80	10	10
Rigid filled hole		80	10	10
Open hole (C)	Multiaxial loading	25	25	50
Al. filled hole		25	25	50
St. filled hole		25	25	50
Rigid filled hole		25	25	50
